# Tamoxifen Citrate Containing Topical Nanoemulgel Prepared by Ultrasonication Technique: Formulation Design and In Vitro Evaluation

**DOI:** 10.3390/gels8070456

**Published:** 2022-07-21

**Authors:** Mohammad H. Alyami, Hamad S. Alyami, Abdullah A. Alshehri, Wijdan K. Alsharif, Ibrahim Ahmed Shaikh, Thamer S. Algahtani

**Affiliations:** 1 Department of Pharmaceutics, College of Pharmacy, Najran University, Najran 66462, Saudi Arabia; mhalmansour@nu.edu.sa (M.H.A.); tsalqahtany@nu.edu.sa (T.S.A.); 2 National Centre for Biotechnology, Life Science and Environment Research Institute, King Abdulaziz City for Science and Technology (KACST), Riyadh 11442, Saudi Arabia; abdualshehri@kacst.edu.sa (A.A.A.); walsharif@kacst.edu.sa (W.K.A.); 3 Department of Pharmacology, College of Pharmacy, Najran University, Najran 55461, Saudi Arabia; i.ibrahimshaikh09@gmail.com

**Keywords:** Tamoxifen citrate, nanoemulsion, surfactants, nanoemulgel, ultrasonic emulsification, MTT assay

## Abstract

The present study aims to design and develop a nanoemulgel formulation of Tamoxifen citrate (TAM), a water-insoluble, potent anticancer drug, using the spontaneous emulsification method to improve topical delivery, achieve high accumulation at the tumour site, and spare the healthy tissues. The oil-based selection was related to the TAM solubility, while the surfactant and co-surfactant were chosen based on the droplets’ thermodynamic stability and size. Afterwards, a pseudo-ternary phase diagram was built for the most promising formulation using two oils, olive and sesame, with a varied mix of Tween 40 as the surfactant and Trascutol HP as the co-surfactant (S_mix_), by the optimisation of experiments. The nanoemulsion (NE) formulations that were prepared were found to have an average droplet size of 41.77 ± 1.23 nm and 188.37 ± 3.53 nm, with suitable thermodynamic stability and physicochemical properties. Both olive and sesame oils are natural food additives due to their associated antioxidant effects; therefore, they showed no toxicity profile on breast cell lines (MCF-7, ATCC number HTB-22). The TAM-NE preparations revealed a prolonged and doublings superior cumulative percentage of in vitro release of TAM compared to TAM plain gel suspension over 24 h. The release data suggested that the Higuchi model was the best fitting kinetical model for the developed formulations of NE1, NE9, and NE18. The extended release of the drug as well as an acceptable amount of the drug permeated TAM via nanogel preparations suggested that nanoemulgel (NEG) is suitable for the topical delivery of TAM in breast cancer management. Thus, this work suggests that a nanogel of TAM can improve anticancer properties and reduce systemic adverse effects compared to a suspension preparation of TAM when applied in the treatment of breast cancer.

## 1. Introduction

Cancer is a disease with a complex, uncontrolled process [1]. It is a leading cause of death worldwide, causing ten million deaths in 2020. However, today, breast cancer (BC) is the most common cancer type, accounting for 2.26 million cases in 2020 [2]. BC is defined as the uncontrolled growth of malignant cells that start in any breast tissue and develop over time. In 2020, the international agency for cancer research reported that the number of deaths related to BC exceeded 685,000, while 7.8 million women have been diagnosed with BC over the last five years, which threatens women’s lives worldwide [2]. Additionally, there are various types of BC, including ductal carcinoma, which originates in the cells coating the ducts that deliver milk to nipples and accounts for >75% of BC. Lobular carcinoma originates in the milk-secreting glands of the breast and reflects similar behaviour to ductal carcinoma. Other types can be located in different areas in the breast, such as the skin, fat, and connective tissues [3].

**Figure 1:**
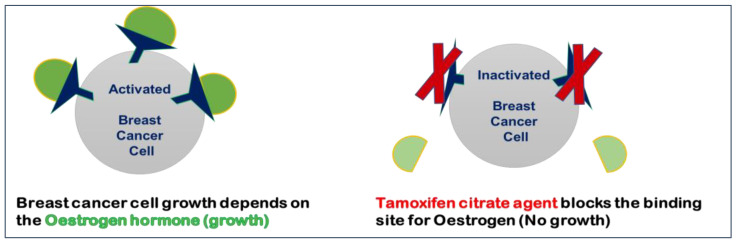
Tamoxifen is capable of blocking the oestrogen hormone receptor on the breast cells, thereafter, cancer cells cannot grow when they are not activated.

Tamoxifen citrate (TAM) is a lipid-soluble drug that is extensively used as first-line chemotherapy and for the inhibition of oestrogen receptors in BC (Rehnmark et al., 2022). TAM is a selective oestrogen receptor modulator that inhibits oestrogen receptor (ER) transcriptional activity by binding to oestrogen receptors. Hence, it helps block proliferation and induces apoptosis of BC cells, as shown in Figure 1. Nevertheless, the pharmacologic anticancer activity of TAM is associated with its capability to contest with oestrogen on ER receptors after administration, and therefore, TAM can block the oestrogen action and prevent cancer cell growth [4].

However, several problems related to the oral administration of TAM drugs, such as lack of specificity and cytotoxicity, may hinder the patient’s adherence or stop its use [1]. This involves but is not limited to strokes, difficulty in speaking, understanding, and seeing, dizziness, severe headaches, and a high risk of having cataracts. Moreover, it is also associated with blood clots in the lungs, chest pain, breath shortness, confusion, tingling or numbness of the face, arms, or legs, cancer of the uterus, pain or pressure in the pelvis, leg swelling or tenderness, and abnormal vaginal bleeding. Despite all this, the TAM drug remains one of the top-listed prescribed anticancer agents in the management of BC, either to reduce the risk of developing serious BC types or to reduce the risk of BC in women who are at high risk because of their age and family history [5].

Thus, novel delivery systems such as emulsions can increase the lipid-soluble drugs in water and offer extra benefits via skin barriers such as enhanced permeability [6]. Studies have shown that emulsion systems offer a suitable carrier for poorly water-soluble drugs such as amiodarone and paclitaxel [7,8]. Consequently, the nanoemulsion (NE) system may help to reduce TAM systemic adverse effects and increase TAM efficacy once applied transdermally. NE is regarded as a good carrier for a wide range of pharmaceuticals due to its great stability and high permeation properties [9,10]. In addition, NE can offer a prolonged release of the drug load [11]. NE is often prepared from a monolayer of phospholipids formed from surfactant and vegetable oil dispersed in water with a droplet size of <100 nm [6]. 

Unlike typical dispersion preparations, which are tenfold larger when measured in micrometers, NE carriers were reported to increase bioavailability and enhance the efficacy of several pharmaceuticals as inflammatory inhibitors [12], including propranolol [13], econazole nitrate [14], lapachol [15], terbinafine hydrochloride, [16] and Ibuprofen [17]. Furthermore, the conversion of NE formulations into a nanoemulgel (NEG) preparation is a favourable approach that can enhance the pharmaceutical properties of the final product, such as its spreadability and stability. In addition, the contact of the product with the skin and the release period of the active ingredient become longer [18]. 

Therefore, the present study aimed to prepare and characterise TAM-containing NEG formulations suitable for topical applications in BC. NE can increase the solubility of TAM and enhance its penetrability through the skin after topical administration. It will also help to reduce TAM toxicity after oral administration and increase its bioavailability at the tumour site [19].

## 2. Results and Discussion

### 2.1. Screening of NEG Components—A Solubility Evaluation of TAM

It is important to examine the effects of different components’ behaviours when formulating NEs. High drug solubility in the oil phase is vital for developing an NE system as it permits the lipid carrier to encapsulate higher drug concentrations. The specific gravity and HLB value for the oil phase are also significant in the development of the NE system [20].

**Figure 2:**
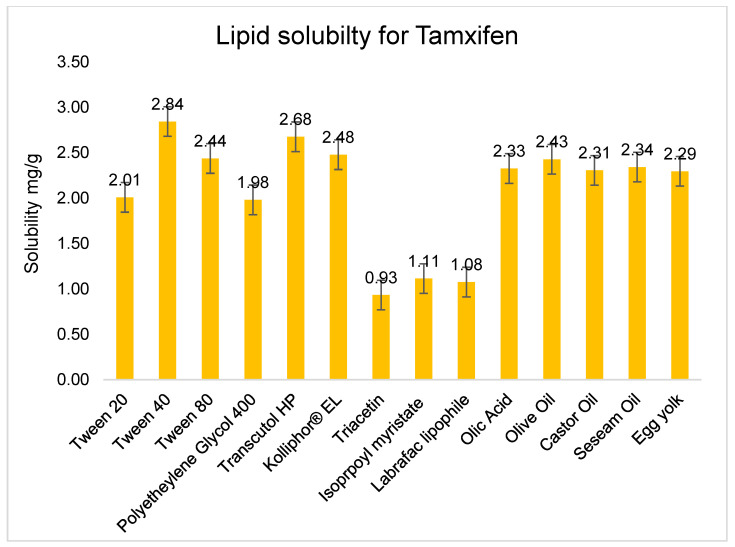
Solubility data of Tamoxifen agents in various formulation excipients; data are expressed as mean ± SDD (*n* = 3).

Figure 2 shows that the solubility of TAM was investigated in different oils and surfactants such as oleic acid, castor oil, olive oil, sesame oil, Tween 20, Tween 40, Tween 80, PEG 400, Transcutol HP, Kolliphore EL, triacetin, isopropyl myristate 98%, and Labrafac lipophile. From various studies on the components and the data available, the maximal solubility of TAM was found in olive and sesame oils, and in Tween 40 as a surfactant and in Transcutol HP as a co-surfactant. The high solubility in sesame and olive oil may be ascribed to the long-chain triglycerides, which helped in the maximal solubilisation of TAM [20,21]. However, Tween 40 exhibited maximum solubility compared to other examined surfactants, which are yellow to orange viscous liquids or pastes at room temperature, with a high HLB value of 15.6, which reflects high solubility in water, ethanol, methanol, ethyl acetate, and acetone. This can also be associated with molecular weight, as Tween 40 has a reduced particle size compared with other large polymeric surfactants. This can benefit the micellar solubilisation enhancement of the lipid drug in the oil phase and enables quicker emulsification efficiency to produce a fine NE system. Likewise, Transcutol HP revealed the highest solubility measurements for TAM. Transcutol HP is considered a very good co-surfactant and solubiliser for compounds that are hydrophobic and have low percutaneous transport [22]. Additionally, the use of Transcutol HP can be further attributed to an increase in the drug loading and fluidity of the preparation, which will be helpful in fast emulsification and attaining a small globule size [23].

### 2.2. Preformulation 

Based on the solubility studies displayed in Figure 2, olive oil and sesame oil possessed the best oil base for the highest TAM solubility, with 2.34 + 0.05 mg/g and 2.43 + 0.06 mg/g, respectively. According to the literature, both oils are enriched with vitamin E, which helps to protect skin cells from the destruction caused by environmental factors such as pollution, UV rays, and many other toxins [24,25]. Further, because diseases are caused by the absence of certain basic nutrients, a lack of vegetable oils may lead to serious health issues like cardiovascular disease, obesity, cholestasis, and other related diseases [26]. 

From a stability perspective, olive and sesame oils are considered stable when it comes to oxidation; however, they contain low levels of ω3 fatty acids [26]. Olive oil comprises a fatty acid and triacylglycerol composition, phenolic compounds, and phytosterols, increasing stability under heat and deep-frying conditions [1,26]. Likewise, sesame oil comprises high quantities of tocopherols, sesamolin, and sesamin lignans, which improve oxidation stability and also provide health-promoting effects, such as anti-proliferative activity on cancer cells and anti-inflammatory effects [26,27,28,29].

Additionally, the study used well-established non-ionic surfactants generally recognized as safe (GRAS), namely Tween 40 and Transcutol HP, for an enhanced emulsification process in the development of TAM-NE systems [18,30,31]. Therefore, the applicability of several S_mix_ was scanned by preparing seven different ratios of Tween 40 and Transcutol HP, as follows: 1:2, 2:1, 3:1, 4:1, 1:2, 1:3, and 1:4. The phase behaviours of NE pre-formulations contain these various components. Transcutol HP can enhance the formulation developed for the TAM-NE system as it works as a co-surfactant and a penetration enhancer through the skin barrier upon application [32,33].

### 2.3. Preparation of NE Using an Ultrasonication Technique

According to the data from the preformulation study, the oil phase, S_mix_ phase, and water phase concentrations were analysed, and initially, 49 NE formulations were prepared for investigation as shown in Table 1. Afterwards, following the analysis, out of these 49 formulations, only 23 NE formulations were selected as stable and underwent further investigation and analysis. It is worth mentioning that those ratios with a greater percentage of hydrophilic surfactant (Tween 40) as compared to lipophilic co-surfactant (Transcutol HP), were regarded due to the lipophilic nature of the drug and the possibility of oil in water (o/w) emulsion. The results are shown in Table 1. A high-energy ultrasonication technique was employed to prepare the TAM-loaded NE system, as previously reported in [9,30,31].

The minimum percentage of the oil used was 20.0%, as shown in Table 1 above, to increase the amount of TAM drug dissolving and decrease the potential of low drug loading. Meanwhile, the maximum amount of water used was 40.0%, as above this ratio the surfactant percentage may increase by over 40%. Accordingly, the best concentration of the S_mix_ in each formulation was selected, and it remained constant to achieve the highest possible drug loading (mg/mL) in each NE system. Firstly, the uniform dispersion system was prepared by mixing 1% *w*/*w* (10 mg/g) of TAM drug in the mixture of the oil phase and S_mix_ phase at the ratios presented in Table 1, through the vortexing technique. Hence, the aqueous phase was added with continuous vortexing for one minute [34]. Afterwards, the uniform dispersion system obtained was ultrasonicated using an ultrasonic homogenizer (FS-300N, Gaoyou city, China) in a water bath for a varying time duration (five minutes) at a constant amplitude of 50% [16,18]. The impact of the composition variables on the droplet size and PDI of the developed TAM-NE system was evaluated, as shown in Table 2.

### 2.4. Characterisation of the TAM-NE Formulations That Were Prepared

All the successful TAM-NE formulations (around 23) that were developed were exposed to thermodynamic stability testing, which included heating-cooling cycles, centrifugation tests, and freeze-thaw cycles. However, studies revealed that only three of the developed formulations (NE1-NE9-NE3) revealed emulsion stability (no creaming, cracking, or coalescence) and were successfully accepted for the stress tests, indicating that the NE system prepared was in a state of acceptable dispersion. Droplet size has a significant effect on the transdermal transportation of NEs [35]. Those formulations that passed the thermodynamic stability tests and were within 100 nm or less in size were kept and carried over for further tests, as in Table 3. The three previously mentioned formulations were completely transparent in appearance, with a small droplet size < 100 nm. It is significant to note that NEs differ from microemulsions (MEs), since MEs are referred to as thermodynamically stable isotropic liquids made by mixing oil, water, and surfactants [36]. In practice, NEs might be unstable when compared to MEs due to droplet coalescence. Appropriate surfactants are necessary for facilitating the preparation of a stable system and guaranteeing its kinetic stability during its shelf life. According to McClements (2012) [36], a combination of S_mix_ is preferred over a single surfactant to form a stabilised NE system.

The droplet size, ZP, and PDI are the key features that are fundamentally measured when designing and developing a stable NE system. Such factors of NE determine their bulk properties, product performance, stability, and appearance [30,33,37]. Hence, optimised formulations of TAM-NE were characterised for a mean droplet size, PDI, ZP, and percentage of TAM-load, and the results are shown in Table 4.

The study results revealed that 25:40:35 was the optimised ratio for oil, S_mix_, and water in terms of droplet characteristics. However, changing the base oil or S_mix_ ratio used showed a significant impact on droplet measurements (*p* < 0.05). 

This was obvious between emulsions developed NE1 and NE18, which were prepared from a similar percentage of (oil:water mix) (Table 2) in addition to the S_mix_ (1:3); however, the results exhibited significant differences between both formulations. This is attributed to the change in the NE oil base from olive to sesame by a factor of 1.73. However, when the percentage of the lipophilic surfactant was increased by 2:1 in NE18, the mean droplet size was decreased to 29.65 + 2.6 nm compared to 55.25 + 2.8 nm, while maintaining the oil concentration (25%) and TAM drug loading constant (4 mg/mL).

**Figure 3:**
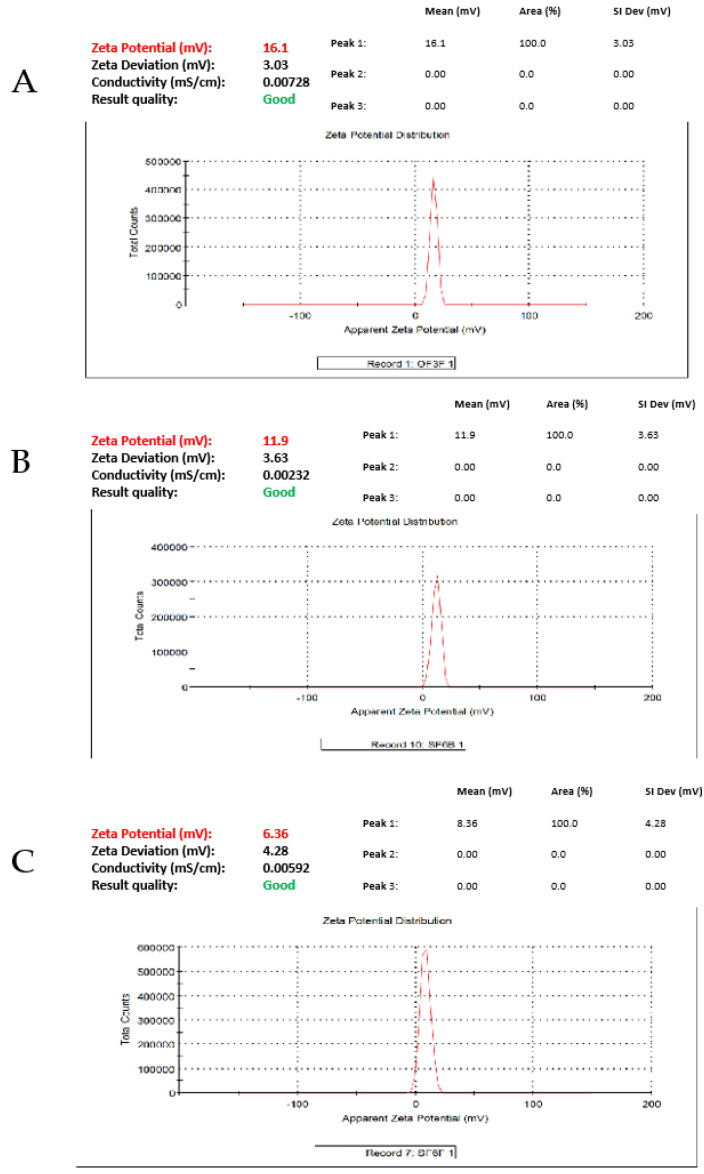
Zeta potential distribution of the optimised TAM-NE: (**A**) for NE1, (**B**) for NE9, and (**C**) for NE18.

Likewise, the ZP and PDI results showed significant variations when the oil base or S_mix_ ratio was changed (*p* < 0.05) (Figure 3). The developed TAM-NEs exhibited stable systems with considerable encapsulation efficiency (>97%) and an agreeable drug-loading capacity (>2 mg/mL) [38]. This confirms that the composition and percentage in each formulation system can greatly influence the size distribution of NE. Considering this, the NE1, NE9, and NE18 formulations were selected for use in the in vitro drug release study.

### 2.5. In Vitro Drug Release of the Optimised TAM-NEs

**Figure 4:**
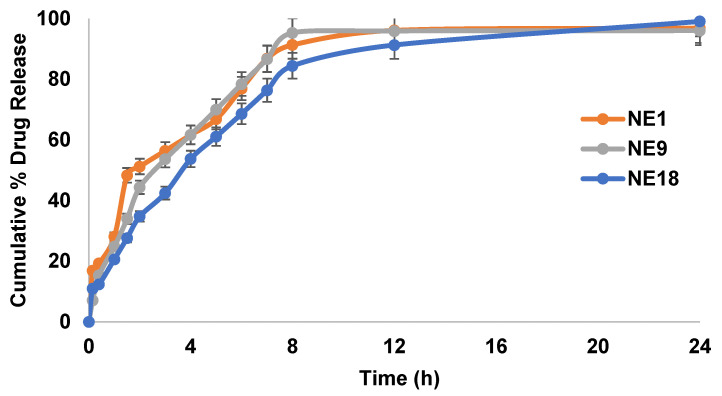
In vitro release of TAM from the TAM-loaded NE (nanoemulsion) system; data represent mean ± SD (*n* = 3).

The in vitro drug release experiment was conducted for 24 h in PBS (pH = 5.5), as shown in Figure 4. TAM-NE followed typical biphasic drug release behaviour for the emulsion system. Initially, the studied TAM-NE formulations revealed a burst release, followed by a controlled release. The data in Figure 4 present TAM’s in vitro release behaviour from the optimised formulations (NE1, NE9, and NE18) over 24 h. All the investigated TAM-NE formulations were capable of releasing over 90% of the drug encapsulated within the first 12 h. This quick release of the TAM-loaded NE might result from the drug located near the surface of the NE system, as they normally display a big initial burst release. However, those with uniformly loaded drugs could provide a sustained drug release over time. In addition, small particle sizes usually offer a larger surface-to-volume ratio, which pushes the rapid release [39]. This can confirm that the mean droplet size of the NE system plays a determinant role in the in vitro drug release, irrespective of the percentage (%) compositions of the NE system. The study results are in agreement with some published studies [8,18,34]. The second sustained phase of TAM-loaded NE was recorded for the next 24 h. By the end of the 24-h period, the maximum percentage of cumulative TAM released from the tested NE formulations was 96–99.1%. 

Additionally, the release and diffusion characteristics of the TAM drug from the optimised NE release were applied to various kinetic models, including zero order, first order, the Higuchi model, and Korsmeyer–Peppas in Table 5. The release kinetics of all the TAM-NEs (NE-NE9-NE18) were determined from the cumulative amount of drug released vs. the time profile. The best-fitting release kinetics were selected by the high value of the correlation coefficient (r^2^). Overall, the best curve fitting showed that TAM released from the optimised TAM-NEs follows the Higuchi model, as presented in Table 5. These models explain the release pattern to be dependent on some individual property. To explain, the diffusion of the drug from the NE system to the receptor medium becomes the rate-limiting step of the drug release after a while. The release of TAM-NEs was modest since the three optimised formulations have similar release profiles. All three optimised formulations were approved to be converted to NEG.

### 2.6. Preparation and Characterisation of the TAM-NEG System

The low viscosity of TAM-NEs and their liquid properties make NE formulations difficult to administer topically. Thus, the optimised formulations (NE1-NE9-NE18) were dispersed in the Carbopol^®^ 940 gel matrix to make the final concentration of 0.5% (*w*/*w*) of the TAM drug into a NEG system, with the preferred consistency for patient-friendly topical application. Gelation was achieved by adding triethanolamine (TEA) to the Carbopol dispersion. The obtained hydrogel system was kept at rest overnight before use. For an appropriate topical gel application, the formulation must have sufficient mechanical strength and the ability to develop and retain a gel form, which is vital for good spreadability and small drainage of the formulation. The gel strength for topical application should be between 25 and 50 s, as <25 s cannot maintain its consistency and may quickly drain away, whereas >50 s would be stiff, causing difficulty when spreading the product topically [40]. The gel strength of the TAM-NEGs system was found to be 41.66 + 1.53 s, 48.77 + 0.58 s, and 46.33 + 2.08 s, respectively, while the placebo gel was 41.66 + 1.53 s; hence, the results were similar. 

The spreadability coefficient for the developed TAM-NEG system and placebo gel systems was investigated, as shown in Table 6. The pH range of the TAM-NEGs was acceptable for topical application as it is close to the pH of the skin (5.55 + 0.031). This emphasised that the newly developed TAM gel is safe for skin application. The percentage of the TAM content within the optimised TAM-NEGs was also measured using UV-spectrophotometric analysis and was found to be >96% + 0.26. The results are depicted in Table 6. 

**Figure 5:**
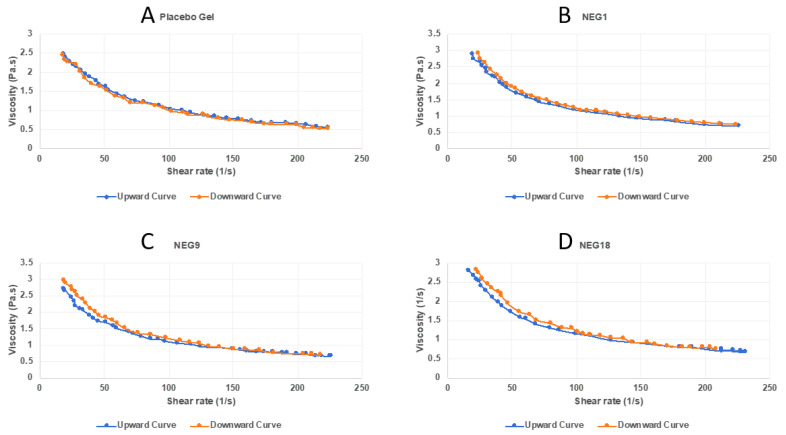
Rheology profile of (**A**) Placebo gel, (**B**) TAM-NEG1, (**C**) TAM-NEG9, and (**D**) TAM-NEG18.

The rheological profile of the developed TAM-NEGs and the placebo gel of Carbopol^®^ 940 of the same concentration (0.5% *w*/*v*) are demonstrated in Figure 5. It is clear that the placebo gel and the TAM-NEGs have similar rheological behaviours, and the incorporation of TAM inside a NEG system did not affect its rheology behaviour.

All investigated gel preparations underwent a gel-to-sol transformation, displaying shear thinning under the influence of shear stress as a result of the loss of close packing and breakdown of the inter-droplet network for repulsive and attractive nanogels, respectively [36,41]. It was clear that the viscosity of TAM-NEG and the placebo gel reduced when the applied shear rate was increased and vice-versa (illustrated through the downward and upward curves in Figure 5). This indicates a non-Newtonian, pseudoplastic property with thixotropic behaviour for developed TAM-NEGs [42,43]. This is appropriate behaviour for pharmaceutical formulations designed for topical skin treatment [34,44,45].

### 2.7. Ex Vivo Skin Permeability Study

**Figure 6:**
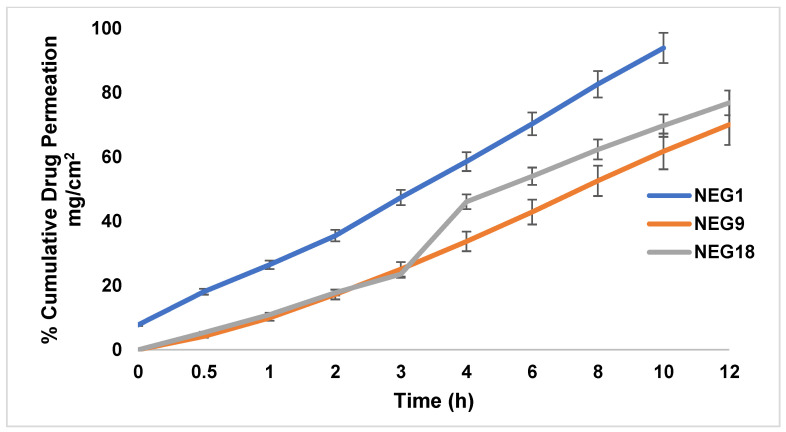
In vitro cumulative percentage of drug permeated via rat skin from different optimised TAM-NEG (NEG 1, NEG 9, NEG 18).

The ex vivo drug deposition study was conducted using Franz diffusion cells on the excised skin of albino rats for the three developed TAM-NEG systems. The results obtained from the ex vivo skin permeability are depicted in Figure 6. 

The pH values for the developed NEG1, NEG9, and NEG18 were about 5.52 + 0.023, 5.51 + 0.021 and 5.48 + 0.022, respectively. All preparations maintained high drug content uniformity of 97% + 0.39, 96% + 0.44 and 99% + 0.41, respectively. The results are presented in Table 7. 

For drug permeation, the results in Table 8 show that the cumulative amount of TAM drug permeated through the skin was 915.8 + 2.57, 796.24 + 2.11, and 2880.9 + 2.52 μg/cm^2^, respectively, from the TAM-NEGs prepared, whereas the amount of TAM deposited on the skin was 796.24 + 2.1, 915.8 + 2.57 and 864.44 + 1.65 μg/cm^2^, respectively. The amount of TAM drug permeating the deep skin layers was almost three times as high as TAM retained in the skin (Table 8). The TAM’s percutaneous drug flux (Jss) from the TAM-NEG preparations was high (53.16 + 3.21, 51.02 + 4.13, and 68.59 + 4.41, respectively).

Further, a noteworthy increase (*p* < 0.05) in the percutaneous drug flux was observed of TAM from TAM-NEGs (53.16 + 3.21, 51.02 + 4.13, and 68.59 + 4.41) compared to the drug flux of TAM from the plain TAM gel (4.39 + 0.50). Similarly, the permeability coefficient (K_10X3) of the TAM drug increased approximately five-fold from the TAM-NEGs (4.25 + 0.11, 4.82 ± 0.09 and 5.48 ± 0.20) when compared to the TAM gel (0.96 + 0.01). Additionally, the permeation enhancement ratio (ER) of TAM from the TAM-NEGs was high at 10.95 + 0.78, 8.03 + 0.55 and 5.71 + 0.78, whereas the local accumulation efficiency (LAE) of the TAM drug was <0.42 + 0.03 from the TAM-NEGs, and for the TAM placebo gel, it was higher (0.89 + 0.12).

The ex vivo studies revealed that the high skin permeability of TAM from the TAM-NEGs can be attributed to the encapsulation of TAM drugs within an NE system. The encapsulation of the TAM drug inside small oil droplets facilitated the enhanced permeability of TAM via the skin, which was observed from the cuts in the lag time for permeation [45]. The lag time (hour) for TAM released from the TAM-NEGs reduced to 0.46 + 0.02, 0.51 + 0.03 and 0.58 + 0.03 compared to 1.65 + 0.47 of the released TAM from the plain TAM gel. 

Further, the presence of Tween 40 in the S_mix_ when developing NE systems as a surfactant can enhance the dermal delivery of drugs, which might have helped in the enhanced skin-penetration of TAM from newly developed TAM-NEGs compared to plain gel [46]. Tweens are capable of altering the skin barriers by inserting them within the skin stratum corneum and creating a strong interaction with water content, which can disturb the skin’s lipid and protein permeability, whereby the skin deposition and permeation for the loaded drug is improved [39,47]. 

### 2.8. In Vitro Assessment of the Cytotoxicity of TAM-NEGs 

**Figure 7:**
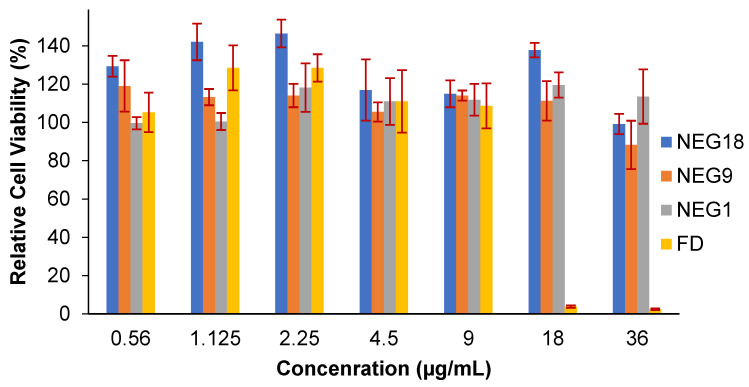
Relative cell viability of three different Tamoxifen-nanogel nanoparticles (NG31,NGE9 and NGE18) and free Tamoxifen Citrate (FD) after their incubations for 24 h with a breast cancer cell line (MCF-7). The data are the result of the MTS assay, which is expressed as relative cell viability (%) and presented as the mean ± SD (*n* = 3), *p* > 0.05.

The in vitro cytotoxicity assessment of the prepared NEGs is an essential step toward biomedical application. In this experiment, increased doses of the free drug and newly developed TAM-NEGs were investigated against the MCF-7 cell line to define the optimal dose that does not cause cytotoxic effects to live tissue and determine ‘safe’ concentrations for future studies. Figure 7 displays the effect of the optimised TAM drug loaded in nanogel formulations (NEG1, NEG9, NEG18) with different concentrations on the cellular metabolic activity of the MCF-7 cell line, which was evaluated using an MTS assay after 24 hours’ incubation time. 

The relative cell viability of the free TAM was also evaluated and compared with the developed NEG systems. The results indicated that high metabolic activity of the human breast cancer cells was achieved in all studied preparations and concentrations applied, even at the maximum concentration used (36 µg/mL). Moreover, the result demonstrated no effect of all applied doses on the cell viability as its level is similar at the lowest and highest concentrations applied (0.56 and 36 µg/mL, respectively). The use of a free Tamoxifen drug exhibited a cytotoxic effect on the MCF-7 cells at a concentration of >9 µg/mL, which caused cell death. The loading of Tamoxifen on the nanoparticle delivery system could reduce the cytotoxic effect and increase the cell viability significantly, as shown in the nanogel formulations loaded with 18 and 36 µg/mL. 

It is worth mentioning that the cell viability percentage of MCF-7 cells after their incubation with the NEG preparations was higher than the actual positive control used in this experiment (cells with DMEM only). This might be due to the compositions of NEGs used in this study, which had a proliferation effect on the breast cancer cell line. Further assessment should be done to confirm this observation and explore its mechanism. Hence, the in vitro evaluation revealed that the developed Tamoxifen-NEGs are a safe preparation for topical applications.

## 3. Conclusions

The NE system was designed and prepared to enhance the solubility, skin deposition, and permeation of the anticancer TAM lipophilic agent to obtain the maximal possible therapeutic value to treat BC topically. The formulations were assessed for their ex vivo skin penetrability attributes in albino rats. The encapsulation of TAM in optimised O/W nanoemulsion formulations of droplet size of around 95.73 + 4.3 for olive oil base formulation and down to 29.65 + 2.6 for sesame oil base formulation using the ultrasonic emulsification technique, producing small droplet sizes down to 41.77 + 1.23 nm. A low concentration of surfactant/co-surfactant (S_mix_ 1:3) was employed as it is desirable for transdermal application and achieves greater therapeutic value. The encapsulation efficiency of developed NEs was greater than 96% of TAM loaded. The release kinetics of the TAM drug loaded into NEs followed the Higuchi model and revealed the sustained release profile over 24 h, 99% of the drug was released. However, the in vitro cumulative percentage of drug permeated via rat skin from optimised TAM-NEGs showed significant differences (*p* < 0.05), i.e., olive base formulations had a superior % of drug permeated compared to sesame base formulations in the first 10 h (93% vs. 69%). Hence, it can be concluded that the newly developed TAM-NEGs performed as a promising delivery system in the enhancement of the transdermal efficacy of poorly permeable TAM drugs, particularly for the long-term management of BC, most importantly by eliminating systemic adverse effects caused by oral TAM administration.

## 4. Materials and Methods

### 4.1. Materials

Tamoxifen citrate (TAM) was purchased from Toronto Research Chemicals INC, North York, Canada. Olive oil and sesame oil were obtained from trusted local shops in Najran University, Najran, Saudi Arabia. Trascutol HP and Labrafac PG (propylene glycol dicaprylocaprate) were purchased from Gattefose, Saint-Priest, France. Polysorbate 20, Tween 20, Tween 40, and Tween 80 were obtained from Sigma-Aldrich, Taufkirchen, Germany. PEG 400 (Polyethylene glycol 400) was provided from Merck Schuchardh, Hokenbrunn, Germany. All the other chemicals and materials were of pharmaceutical or analytical grade.

### 4.2. Screening and Optimisation of the Formulation Components 

Oil, surfactant, and co-surfactant are the primary formulation contents used to design an NE system for topical application prepared using low-energy or high-energy emulsification [34]. The maximum amount of TAM drug solubilized in the NE system was investigated to determine the highest amount of TAM loading into the oil phase. Additionally, the solubility of TAM was evaluated in the surfactant/co-surfactant phase (S_mix_), which can be a useful way to predict the maximum loading of the drug concentration in the intended NE system. 

In brief, an additional amount of TAM was added to 3 mL of the selected vehicle in 5 mL capacity closed vials and then mixed well using a vortex mixer. Afterwards, the vials were capped tightly and agitated in a water bath shaker (25 ± 2 °C) for 48 h. The samples were centrifuged for 15 min (5000× rpm), the supernatants were transported and filtered via a 0.22 µm syringe membrane filter (Whatman^®^ Puradisc), and the amount of solubilized TAM was quantified at a wavelength of 235 nm using a UV-visible spectrophotometer [48].

Similarly, the S_mix_ used in this study were assessed upon their respective maximum nanoemulsifying regions. The S_mix_ phase emulsification efficiency was performed as per the reported method by Algahtani et al. (2020), with minor adjustments [49]. The dispersion system of the S_mix_ phase could emulsify the required amount of the investigated oil phase, which allowed it to equilibrate and provided a homogenous milky dispersion system, after a continuous addition of water drops. The results achieved from the drug solubility and emulsification efficiency of the S_mix_ phase formed the foundation for the formulation compositions of the planned TAM-NE system. In this way, the percentage of these oils, S_mix_, and the water phase compositions were finally set.

### 4.3. Preparation of TAM-NE Using a High-Energy Technique

The TAM-NE system was developed using a high-energy ultrasonication method, as described by Algahtani et al. [34]. Briefly, the O/W aqueous dispersion system was prepared through the vortex of a 1% *w*/*w* (4 mg/g) mixture (oil and S_mix_ phase) followed by the addition of the water phase with uninterrupted vortexing for one minute. Afterwards, the produced O/W aqueous dispersion was introduced to ultrasonication with an amplitude of 50%, 300 W, in a water bath for five minutes at a constant for droplet size reduction (Ultrasonic Homogenizer, FS-300N, Gaoyou city, China) [30,50]. To choose the best formulation of the TAM-NE system, several formulations of various ratios were suggested and made for advanced investigations [46].

### 4.4. Characterisation of TAM Nanoemulsion 

TAM-loaded NE formulations were initially made using a high-energy ultrasonication technique, and the formulation of specific characteristics was assessed for thermodynamic droplet size distribution, polydispersity index (Pdi), zeta potential (ZP), stability, viscosity, and the amount of the drug loaded in the formulation. To ensure reproducibility, all experiments were conducted in triplicate (*n* = 3) [6].

### 4.5. Thermodynamic Stability 

Various stress condition tests were applied to the prepared TAM-NE systems, such as heating (4 °C) and cooling cycles (40 °C) and freeze (−21 °C) and thaw cycles (+25 °C), with storage at a specified temperature for 48 h. The stability of the prepared NE systems was assessed by applying centrifugation stress; 1 mL of the system was added to 100 mL distilled water and centrifuged for 30 min (5000× rpm), and phase separation was inspected visually [31,34].

### 4.6. Droplet Size, PDI, and ZP Measurements

The analysis of the average droplet size, PDI, and ZP for the different TAM-NEs that were prepared was determined by the Dynamic Light Scattering (DLS) technique (at 25 °C) using a Zetasizer Nano ZS90 (Malvern Instruments, Malvern, UK) [6,12].

### 4.7. The Viscosity of the TAM-NE System 

The viscosity of the optimised TAM-NE system was studied, with no dilution, using a Bohlin rotational viscometer at ambient temperature (25 °C) [6,12].

### 4.8. Drug Content Analysis

The percentage of TAM loaded in the proposed NE formulations was measured at λmax 235 nm using a UV-visible spectrophotometer [8,34]. A 100 µL sample of TAM-NE was diluted to 1000 times with acetone. 

### 4.9. In Vitro Drug Release Study

The TAM-NE systems, which withstood the stability test and had a small droplet size < 100, namely NE1, NE9, and NE18, were further investigated for their in vitro drug release behaviours using the dialysis bag technique [8,34]. Dialysis bags provided by Merck dialysis sacks (12–14 kDa) were filled with 1 mL of developed TAM-NE (NE1, NE9, and NE18) and subsequently suspended in a phosphate buffer medium of pH 7.4 (37 ± 0.5 °C). At a fixed time interval, 1 mL aliquots were taken and replaced by the same volume of fresh medium. The amount of TAM in the optimised formulations was quantified using UV-spectroscopy (λmax 235 nm). Experiments were carried out in triplicate.

### 4.10. Preparation and Characterisation of TAM-NE in the Nanogel System

Emulsions are liquid and of low viscosity, making them an inappropriate system for topical application. Consequently, forming a NEG system is an ideal solution for this matter. The three optimised RAM-NEG formulations were then dispersed in Carbopol^®^ 940 (0.5% *w*/*w*) gel to form TAM-NEG [16,17]. Glycerin was also used as a humectant for the dispersion system, which helps to provide a smooth and soothing effect (5% *w*/*w*). The dispersion media was neutralised at pH 5.5 using triethanolamine, which was added into the system in a drop manner until the transformation into a hydrogel system was complete. The characteristics of the Newley TAM-NEG system developed were investigated using the same methods previously mentioned [17,34].

### 4.11. Ex Vivo Skin Permeability 

The animal study protocol to conduct ex vivo permeability studies in a Wistar rat was approved by the ethical committee at Najran University, KSA, according to their guidelines (Ref. No: 443-41-10223- DS). The rats were housed under standard conditions (25 + 2 °C and 55 + 5%RH). Polypropylene cages were supplied to house the rats, and a standard laboratory diet and water ad libitum were provided regularly. Wistar rat skin was removed and used for the ex vivo skin permeation experiment. The ex vivo permeability profile across the rat skin was conducted for both the developed TAM-NEG and unmodified TAM gel, as per the method described previously [34,44]. Local accumulation efficiency (LAE) was calculated as a ratio of TAM retained in the skin to that permeated through the rat skin for both the TAM-NEG and unmodified gel systems [45].

### 4.12. In Vitro Assessment of the Cytotoxicity of TAM-NEGs 

The cytotoxicity of different TAM-NEG components (NE1, NE5, and NE7) was assessed in vitro on BC cell lines (MCF-7, ATCC number HTB-22) to determine the optimum doses of applied TAM-NEG that is safe on living tissue, causing a beneficial effect on the treated cells. The MCF-7 cell line was cultured in complete Dulbecco’s Modified Eagle’s Medium (DMEM), to which fetal bovine serum (FBS) and other cell culture media components essential for cell growth were added. The MCF-7 cells were used in this study between passages 4 and 10 [37]. The effect of free TAM and TAM-NEGs on the cell viability of tested cells was evaluated using a colorimetric assay (MTS) that determines the metabolic activity of the cells [50]. 

The MTS kit containing a 3-(4,5-dimethylthiazol-2-yl)-5-(3-carboxymethoxyphenyl)-2-(4-sulfophenyl)-2H-tetrazolium solution was provided by Promega (Southampton, UK) under the name of Cell Titer 96^®^ Aqueous One Solution Cell Proliferation Assay. The fully grown MCF-7 cells, separated from the flask using trypsin, were then grown in 96-well plates with a seeding density of 1.5 × 104 cells/well, and attached to the plate surface overnight at a humidity of 5% CO_2_ cell culture in an incubator at 37 °C. The next day, the cell culture media was aspirated and washed using PBS, and then 100 µL of different doses of the investigated formulations (from 0.56 to 36 µg/mL) were introduced to the MCF-7 cells and incubated for 24 h. The BC cells were incubated with DMEM and 0.1% (*v*/*v*) Triton X-100 solution, which were employed as positive and negative controls. 

The investigated samples were then removed and 100 μL of fresh cell culture media was added to each well, followed by adding 20 μL of the MTS solution. The well-plate was incubated for a further 2–3 h at 37 °C. Subsequently, the absorbance of the MTS solution was measured at a wavelength of 490 nm using the plate reader (Cytation™ 3, BioTek Instruments Inc., Winooski, VT, USA). The equation below was used to calculate the level of cell viability [9,25]:

(1) $$\mathrm{Cell}\;\mathrm{viability}\;\left(\%\right)=\left(S-T\right)\;/\;\left(H-\;T\right)\times 100.$$

S = the absorbance of the cells treated with the different TAM-NEGs.

H = the absorbance of the cells treated with cell culture media (positive control)

T = the absorbance of the cells treated with Triton X-100 (negative control)

### 4.13. Statistical Analysis 

The statistical analysis was carried out using SPSS (version 23 SPSS Inc., Chicago, IL, USA). The study data were analysed through one-way ANOVA followed by Tukey’s multiple comparisons tests. The results were considered statistically significant when *p* < 0.05 [49,50].

## Figures and Tables

**Table 1:** Various ratios were used for the preparation of the initial 49 formulations of TAM in a nanoemulsion (NE) based system. The oils used were olive and sesame.

Code	S_mix_ Ratio (Tween 40)/(Transcutol HP)	Code	% Oil	% S_mix_	% Water
A	1:1	F1	30	40	30
B	2:1	F2	30	50	20
C	3:1	F3	20	40	40
D	4:1	F4	20	50	30
E	1:2	F5	20	60	20
F	1:3	F6	25	40	35
G	1:4	F7	25	50	25

**Table 2:** Particle size analysis of stable 23 TAM-loaded NE out of the first 49-NE formulations prepared. The results are presented as mean ± S.D (*n* = 3).

Code	Oil Base	% Oil	% S_mix_	% Water	Mean Droplet Size (nm)	Mean Polydispersity Index (DPI)
NE1	Olive Oil	25	40	35	41.77 ± 1.23	0.248 ± 0.05
NE2		20	40	40	118.10 ± 2.13	0.297 ± 0.04
NE3		25	40	35	188.37 ± 3.53	0.095 ± 0.02
NE4		20	40	40	168.93 ± 2.13	0.285 ± 0.01
NE5		20	40	40	126.63 ± 3.06	0.088 ± 0.03
NE6		20	40	40	95.73 ± 3.21	0.163 ± 0.02
NE7		20	40	40	111.77 ± 3.44	0.229 ± 0.03
NE8		25	40	35	153.77 ± 4.51	0.844 ± 0.04
NE9	Sesame Oil	25	40	35	73.47 ± 2.09	0.177 ± 0.03
NE10		30	40	30	81.43 ± 1.66	0.071 ± 0.03
NE11		20	40	40	143.87 ± 1.89	0.075 ± 0.01
NE12		25	40	35	132.80 ± 2.44	0.231 ± 0.02
NE13		30	40	30	109.23 ± 3.01	0.155 ± 0.04
NE14		20	40	40	117.67 ± 3.23	0.179 ± 0.02
NE15		25	40	35	32.22 ± 2.56	0.356 ± 0.03
NE16		20	40	40	45.51 ± 2.89	0.324 ± 0.05
NE17		25	40	35	176.23 ± 3.50	0.779 ± 0.02
NE18		25	40	35	55.25 ± 2.21	0.175 ± 0.01
NE19		20	40	40	46.22 ± 1.21	0.332 ± 0.05
NE20		25	40	35	59.26 ± 1.28	0.193 ± 0.03
NE21		20	40	40	102.60 ± 2.43	0.203 ± 0.02
NE22		25	40	35	69.82 ± 2.11	0.168 ± 0.04
NE23		20	40	40	54.26 ± 1.88	0.138 ± 0.01

**Table 3:** Few TAM-NE formulations have achieved acceptable results following a phase behaviour study and their thermodynamic stability. (✓) means that the formulation passes the stability test.

No	Formula	Heating-Cooling	Centrifugation	Freeze-Thaw
NE1	OF3F	✓	✓	✓
NE9	SF6B	✓	✓	✓
NE18	SF6F	✓	✓	✓

**Table 4:** Analysis of particle size, PDI, ZP, and percentage drug load of the optimised NE formulations. The results represent mean ± SD (*n* = 3).

No	Size (nm)	Zeta Potential (mv)	PDI	Calculated Drug Amount Mg/mL	% Encapsulation Efficiency
NE1	95.73 + 4.3	16.3 + 1.4	0.163 + 0.08	2.16 + 0.16	97%
NE9	55.25 + 2.8	12.3 + 2.1	0.178 + 0.90	2.55 + 0.64	96%
NE18	29.65 + 2.6	8.1 + 2.60	0.351 + 0.13	2.41 + 0.23	99%

**Table 5:** In vitro drug release kinetics of TAM-NEs (R^2^ is the correlation coefficient.).

Formulation	Zero Order R^2^	First Order R^2^	Higuchi Model R^2^	Korsmeyer–Peppas Model R^2^
TAM-NE1	0.595	0.272	0.871	0.398
TAM-NE9	0.590	0.317	0.865	0.545
TAM-NE18	0.709	0.365	0.929	0.527

**Table 6:** Characterisation of the placebo gel and TAM- nanoemulgel.

Parameters	Placebo Gel	TAM-NEG1	TAM-NEG9	TAM-NEG18
Gel strength	41.66 + 1.53	48.77 + 0.58	46.33 + 2.08	46.33 + 1.53
Spreadability factor	1.23 + 0.03	1.27 + 0.02	1.29 + 0.03	1.27 + 0.04
Drug content uniformity	-	97% + 0.36	96% + 0.26	99% + 0.15
pH	5.49 + 0.02	5.55 + 0.03	5.56 + 0.03	5.57 + 0.03

**Table 7:** Stability study of TAM-NEGs over 30 days.

Code	PH	Appearance	Viscosity (cp)	Days Drug Content %
NEG1	5.52 + 0.02	+++	2.93 + 0.03	97% + 0.39
NEG9	5.51 + 0.02	+++	2.96 + 0.02	96% + 0.44
NEG18	5.48 + 0.02	+++	2.82 + 0.03	99% + 0.41

+++ = Excellent.

**Table 8:** TAM drug deposition and permeation data from three optimised TAM-NEGs and TAM gel.

Cumulative Amount of Drug Permeated (μg/cm^2^)	Cumulative Amount of Drug Permeated (μg/cm^2^)	Drug Deposited in Skin (μg/cm^2^)	Lag Time (h) ^a^	Flux ^b^ (μg/cm^2^ h)	Permeability Coefficient ^c^ (Kp × 10^−3^)	Permeation Enhancement Ratio (ER ^d^)	Local Accumulation Efficiency (LAE) ^e^
NEG1	2232.8 ± 3.24	796.24 ± 2.11	0.46 ± 0.02	53.16 ± 3.21	4.25 ± 0.11	10.95 ± 0.78	0.36 ± 0.02
NEG9	2143.1 ± 3.34	915.8 ± 2.57	0.51 ± 0.03	51.02 ± 4.13	4.82 ± 0.09	8.03 ± 0.55	0.42 ± 0.03
NEG18	2880.9 ± 2.52	864.44 ± 1.65	0.58 ± 0.03	68.59 ± 4.41	5.48 ± 0.20	5.71 ± 0.78	0.29 ± 0.02
TAM Gel	516.90 ± 1.95	219.47 ± 2.36	1.65 ± 0.47	4.39 ± 0.50	0.96 ± 0.01	-	0.89 ± 0.12

^a^ The time required to reach a steady state is called the lag time. ^b^ The percutaneous drug flux (Jss) = Amount of TAM permeated/Area x Time. ^c^ Permeability coefficient Kp = Flux/C0 (the initial drug concentration in the donor compartment). ^d^ Enhancement ratio (ER) = Flux from TAM-NEGs/Flux from TAM gel. ^e^ Local accumulation efficiency (LAE) is the ratio of the TAM drug accumulated in the skin to that permeated via the skin.

## Data Availability

The data presented in this study are available in the article.
